# Supplementary material for: The stability of covalent dative bond significantly increases with increasing solvent polarity
Source: Nat Commun. 2022 Apr 19;13:2107. doi: 10.1038/s41467-022-29806-3 (PMC9018688; doi:10.1038/s41467-022-29806-3)
Supplement: Supplementary file 1 — Supplementary Information [file 41467_2022_29806_MOESM1_ESM.pdf]

Supplementary Information

The stability of covalent dative bond significantly increases with increasing solvent polarity

P. Hobza et al.

SUPPLEMENTARY METHODS

Calculations

The geometries (H_3NBH_3, Me_3NBH_3, H_3NBF_3, Me_3NBF_3 and Me_3PBH_3) were optimized using the def2-QZVP basis set by Cuby4 framework[1,2] using Turbomole 7.3 program.[3] The other systems were optimized in Gaussian 09.[4] C_{18} complex was optimized in ωB97XD/def2-TZVPP level of theory. Raman spectra and NMR coupling constant calculations were performed using Gaussian 09. From the calculated Raman intensities smooth spectra were generated by a convolution with Lorentzian bands of 10 cm^{-1} bandwidth. The spectra are in arbitrary units, as the absolute intensities are not measured. The modes of potential energy distributions (PED) are calculated using VEDA software.[5] The CCSD(T) calculations were performed using Molpro 2010.1 version.[6]

Molecular dynamics trajectories were simulated by ORCA 4.2.1 code[7] and visualized by VMD 1.9.2 visualization software.[8] All the simulations were performed at the PBE0-D3BJ/6-31G* level of theory. The simulation time was performed up to 5 ps for calculations involving solvents and 16 ps for gas phase. In the simulation, the step size was set to 1 fs, Berendsen thermostat[9] with time constant of 20 fs was employed to control the temperatures. The temperature for all the simulations was set at 300 K. Selected frames from the trajectories for both gas and solvents were then optimized at PBE0-D3/6-31G* level.

It has been observed that the average bond distances of the N–B bond (1.632, 1.621 and 1.616 Å) obtained from selected optimized frames of MD trajectories for both gas phase and explicit solvents ($CHCl_3$ 49 molecules and CS_2 49 molecules) are in agreement with those N–B bond distances optimized in continuum solvent models.

Nuclear magnetic resonance spectroscopy

NMR spectra were recorded on a Bruker AVANCE III 500 MHz NMR spectrometer (^{1}H at 500.0 MHz, ^{13}C at 125.7 MHz) and Bruker AVANCE III 400 MHz NMR spectrometer (^{1}H at 401.0 MHz, ^{11}B at 128.7 MHz) in various solvents. The samples were prepared by dissolving 7–8 mg of the trimethylamine-borane complex in 0.5 mL of the solvent. The ^{1}J(H–B) coupling values were obtained from ^{1}H NMR spectra of the non-labelled sample and the ^{1}J(N–B) coupling values were obtained from ^{11}B spectra of the ^{15}N-labelled sample. The non-labelled Me_3NBH_3 was purchased commercially and the ^{15}N-labelled compound was synthesized as described below.

Synthesis of tris(methyl-d_3)amine-^{15}N-borane

The tris(methyl-d_3)amine-^{15}N-borane was prepared by distilling tris(methyl-d_3)amine-^{15}N on neat borane dimethyl sulfide complex avoiding the use of any extra solvent (Supplementary Note 1). After sublimation desired product was isolated in good yield and used for further experiments.

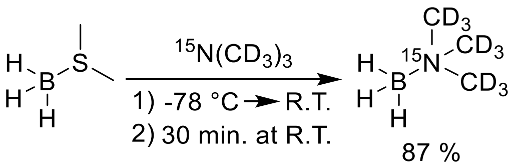

Supplementary Note 1.

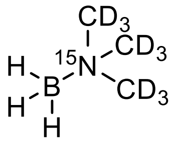

Tris(methyl-*d*$_3$)amine-^{15}*N*-borane. The borane dimethyl sulfide complex (1.3 g, 17.35 mmol, 1.2 eq.) was injected into a 25 mL dried two-neck flask with cold finger adapted on it. The flask and the cold finger were cooled to −78 °C using dry ice/acetone mixture. Tris(methyl-*d*$_3$)amine-^{15}*N* (1.0 g, 14.46 mmol, 1 eq.) was condensed from a cylinder on the solidified borane dimethyl sulfide complex. The cooling bath was removed allowing slow warming up of the reaction mixture (cold finger was kept cold during this process). After stirring for 30 minutes, the white solid content of the two-neck flask was transferred to a 25 mL flask and product was sublimed using a Kugelrohr apparatus (40 °C, 2 mbar, 1 h) with the receiving flask cooled with liquid nitrogen. The product was obtained as a white crystalline material (1.04 g, 15.53 mmol, 87 %).

Mp 93.0–95.4 °C. ^{1}H NMR (400 MHz, Benzene-*d*$_6$): δ 2.01–2.74 (m, 3H). ^{11}B NMR (400 MHz, Benzene-*d*$_6$): δ −6.35 – −8.69 (m, 1B). ^{13}C{^{1}H} NMR (100 MHz, Benzene-*d*$_6$): δ 51.89–52.95 (m). ^{1}H NMR (400 MHz, $CDCl_3$): δ 1.30–2.01 (m, 3H). ^{1}H{^{11}B} NMR (400 MHz, $CDCl_3$): δ 1.70 (s, 3H). ^{11}B NMR (400 MHz, $CDCl_3$): δ −8.93 (q, 3H, *J* = 97.1 Hz). ^{11}B{^{1}H} NMR (400 MHz, $CDCl_3$): −8.93 (s). ^{13}C{^{1}H} NMR (100 MHz, $CDCl_3$): δ 52.35–53.88 (m). IR (KBr): 2368, 2344, 2314, 2262, 2251, 2115, 2077, 1183, 1150, 1116, 1096, 1062, 942, 817, 753 cm^{-1}. MS, *m/z* (%): 82.2 (100, M−H$^-$), 81.2 (20, M−2H$^-$), 79.1 (25), 69.1 (15, M−BH_3), 67.1 (55), 63.1 (20). HRMS (EI+) *m/z*: [M−H$^-$]$^+$ Calcd for $C_3H_2D_9{}^{15}N^+$ 82.1514; Found 82.1515. Anal. Calcd. for $C_{28}H_{20}O_6$: C, 43.91; H, 14.77; N, 17.07. Found: C, 43.26; H, 14.11 N, 16.48.

Raman spectroscopy

Solutions in D_2O, cyclohexane-*d*$_{12}$, benzene, chloroform-*d* and dichloromethane-*d*$_2$ in the same concentration as for NMR (about 7 mg/ml) were measured in a quartz cell, on a ChiralRaman-2X ROA (Biotools) instrument, using the 532 nm laser excitation, laser power was ~100 mW, accumulation times ~20 min. Using our software spectra of pure solvents accumulated at the same conditions as for the solutions were subtracted, and peak maxima read. Note that the instrumental resolution is 3–7 cm^{-1}, and except for D_2O the solvents gave large signals that could be subtracted only partially. In particular dichloromethane-*d*$_2$ was practically immeasurable on this spectrometer, because of a high fluorescence background. Occasionally, to read the peak positions, polynomial baseline was fit to the spectra. The instrumental range is approximately 100–2300 cm^{-1}.

Alternatively, within ~50–3500 cm^{-1}, the dichloromethane-*d*$_2$, chloroform-*d*, and D_2O solution spectra were measured on a Witec confocal Raman microscope, with ~25 mW laser power, 10×

magnification objective, in a glass cell (~0.1 ml). Surprisingly, the fluorescence of the dichloromethane-d_2 solution was lower here and the solute bands could be extracted. As before, pure solvents were used as a baseline, with minor polynomial correction.

Vibrational frequencies

Supplementary Fig. 6 illustrates the linear dependence of the atomic coordinates of Me_3NBH_3 on the stretching distortion If neglecting all the nonlinear terms in this dependence, μ_{ss} becomes constant, simplifying thus markedly the dynamical calculations. Taking into account the limited accuracy of the calculated potential energy function and mainly the practical impossibility to account explicitly for the aggregation effects of the molecular environment, which are of the main concern of this study, we consider it appropriate to rely on this simplest approximation. Interestingly, as one can see in Table 2, the rigid-bender predictions are found to be in a reasonable agreement with experiment and only a single-parameter scaling of the rigid-bender reduced masses brings this agreement more or less within the estimated experimental error bars for all probed solvents.

Supplementary Table 1. The experimental one-bond B–N and B–H indirect coupling values in Me_3NBH_3 complex dissolved in various solvents.

Solvent	ε	^1J(B–H)	^1J(B–N)
Cyclohexane-d_{12}	2.0	99.9	5.3
Benzene-d_6	2.3	98.3	6.2
Toluene-d_8	2.4	98.5	
Chloroform-d	4.7	97.1	[a]
Dichloromethane-d_2	8.9	97.2	7.0
Acetone-d_6	20.5	97.4	6.8
Methanol-d_4	32.6	96.7	
Acetonitrile-d_3	35.7	96.8	7.1
Dimethyl formamide-d_7	37.2	97.0	
DMSO-d_6	46.8	96.7	[a]
D_2O	78.0	94.3	8.5

[a]The coupling value could not be obtained because of signal broadening.

Supplementary Table 2. Observed (ν_{obs}), and calculated harmonic (ν_{harm}) frequencies (in cm^{-1}), the deviation from the experiment ($\Delta\nu$) and assignment of B-N stretching with the potential energy distributions, PED % (C-N stretching in parenthesis) of Me_3NBH_3 in various solvents. The values are calculated at the PBE0-D3/def2-QZVP level.

Solvent	ε	ν_{obs}	PED	ν_{harm}	$\Delta\nu$
cyclohexane	2.0	848	12 (75)	882	34
benzene	2.3	851	12 (74)	884	38
chloroform	4.8	854	15 (71)	885	31
water	78.0	866	18 (67)	888	22

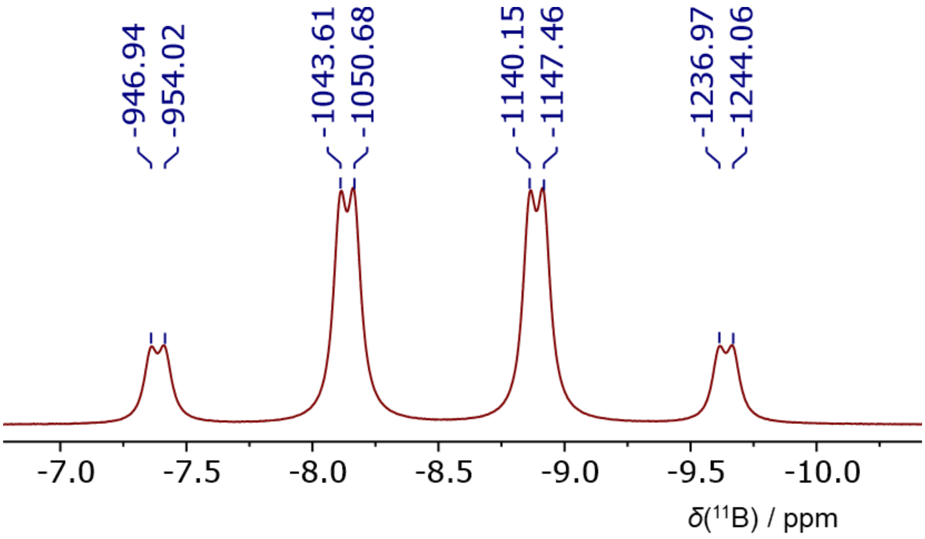

Supplementary Fig. 1 ^{11}B NMR spectrum of ^{15}N-labelled $(CD_3)_3NBH_3$ complex measured in CD_3CN. The peak picking values are in Hz. The signal is a quartet of doublets. The splitting to the quartet is caused by the interaction of boron with three equivalent hydrogen atoms and the splitting to the doublet is caused by the interaction with nitrogen ^{15}N.

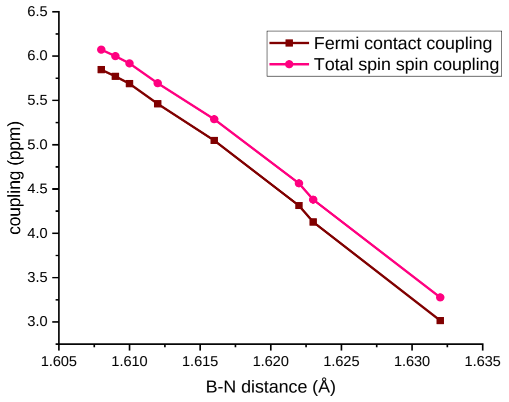

Supplementary Fig. 2 The correlation of total calculated spin-spin coupling and its Fermi contact part with B–N bond distances. The values are calculated at the PBE0/def2-QZVP level.

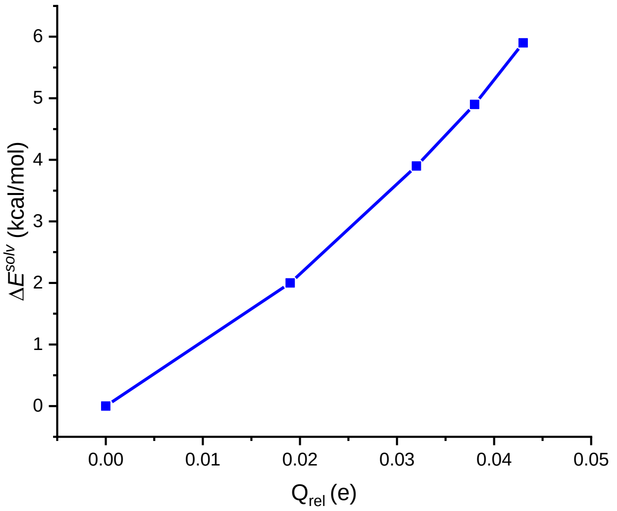

Supplementary Fig. 3 The correlation of change of solvation energy with relative charge transfer. The values are calculated at the PBE0-D3/def2-QZVP level.

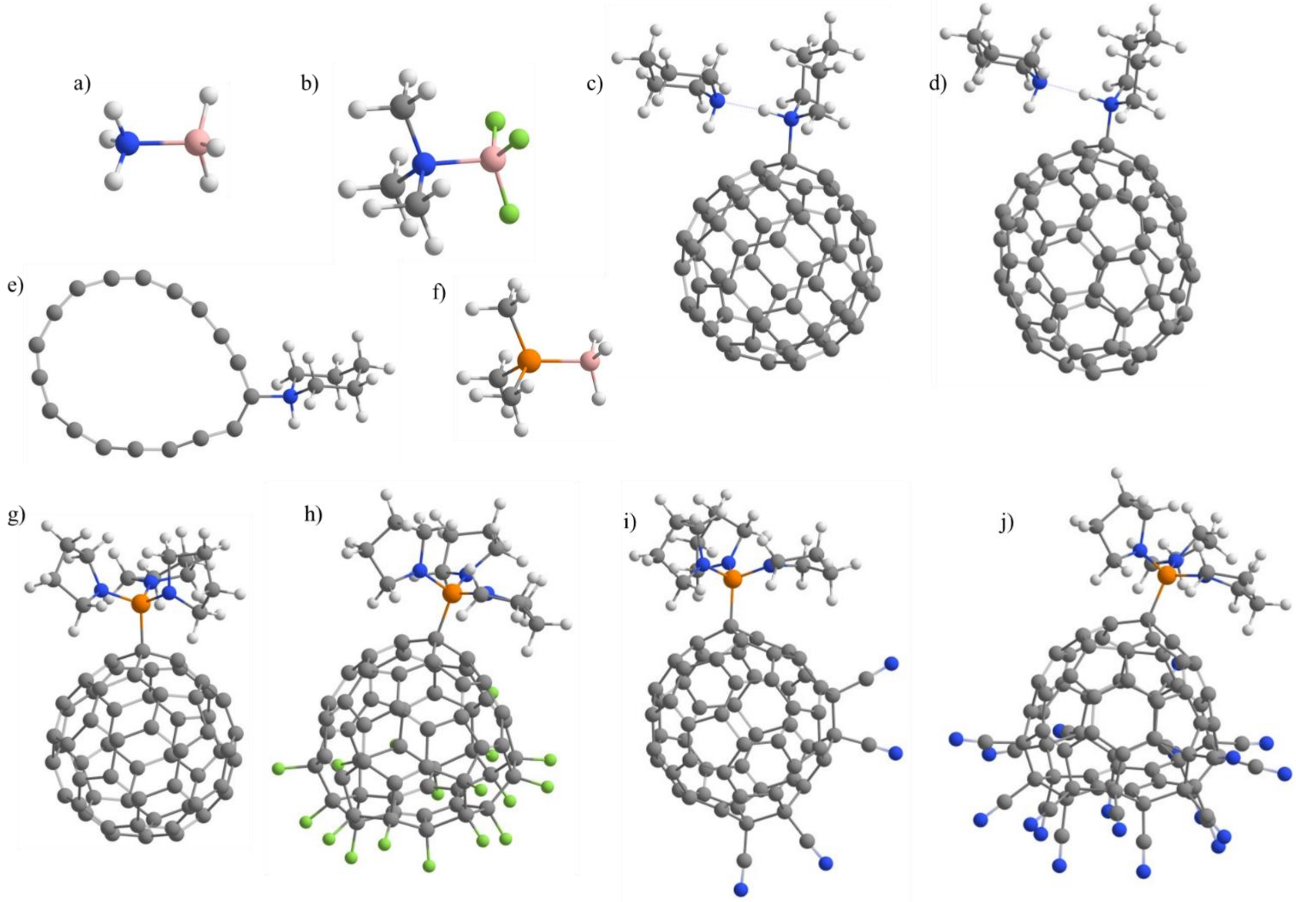

Supplementary Fig. 4 The optimized geometries of various types of dative bond complexes; A) **N→B** : a) H_3B-NH_3, b) F_3B-NMe_3; B) **N→C** : c) C_{60}…pip_2, d) C_{70}…pip_2, e) C_{18}...pip; C) **P→B** : f) H_3B-PMe_3; D) **P→C** : g) C_{60}...P(pyrr)$_3$, h) $C_{60}F_{18}$...P(pyrr)$_3$, i) $C_{60}(CN)_4$...P(pyrr)$_3$ and j) $C_{60}(CN)_{18}$...P(pyrr)$_3$. [C: grey, N: blue, H: white, B: pink, P: orange, F: yellowish green]. The N→B and P→B complexes are optimized at the PBE0-D3/def2-QZVP level, whereas C_{18} complex is computed at the ωB97XD/def2-TZVPP level. The other complexes are optimized at the PBE0-D3BJ/def2-TZVPP level.

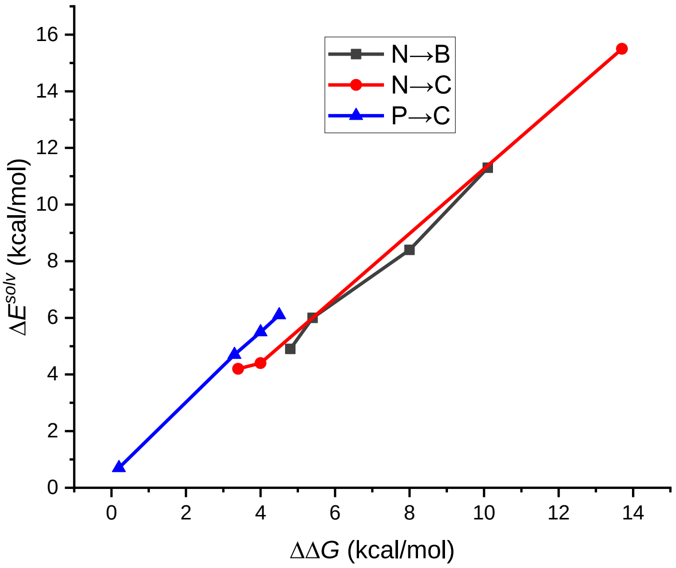

Supplementary Fig. 5 The correlation of $\Delta\Delta G$ and ΔE^{solv} for different types of dative bonds, N→B, N→C, and P→C. For N→B complexes, the values are calculated at the PBE0-D3/def2-QZVP level, whereas for the other complexes, the values are calculated at the PBE0-D3BJ/def2-TZVPP level.

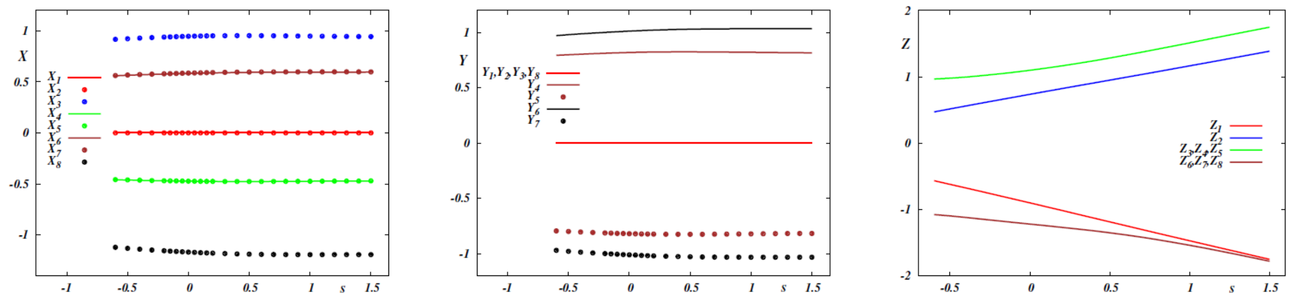

Supplementary Fig. 6 The correlation between the B-N stretch (*s*¸Å*)*, energy minimum pass and cartesian atomic coordinates (X,Y, Z, in Å) of Me_3NBH_3

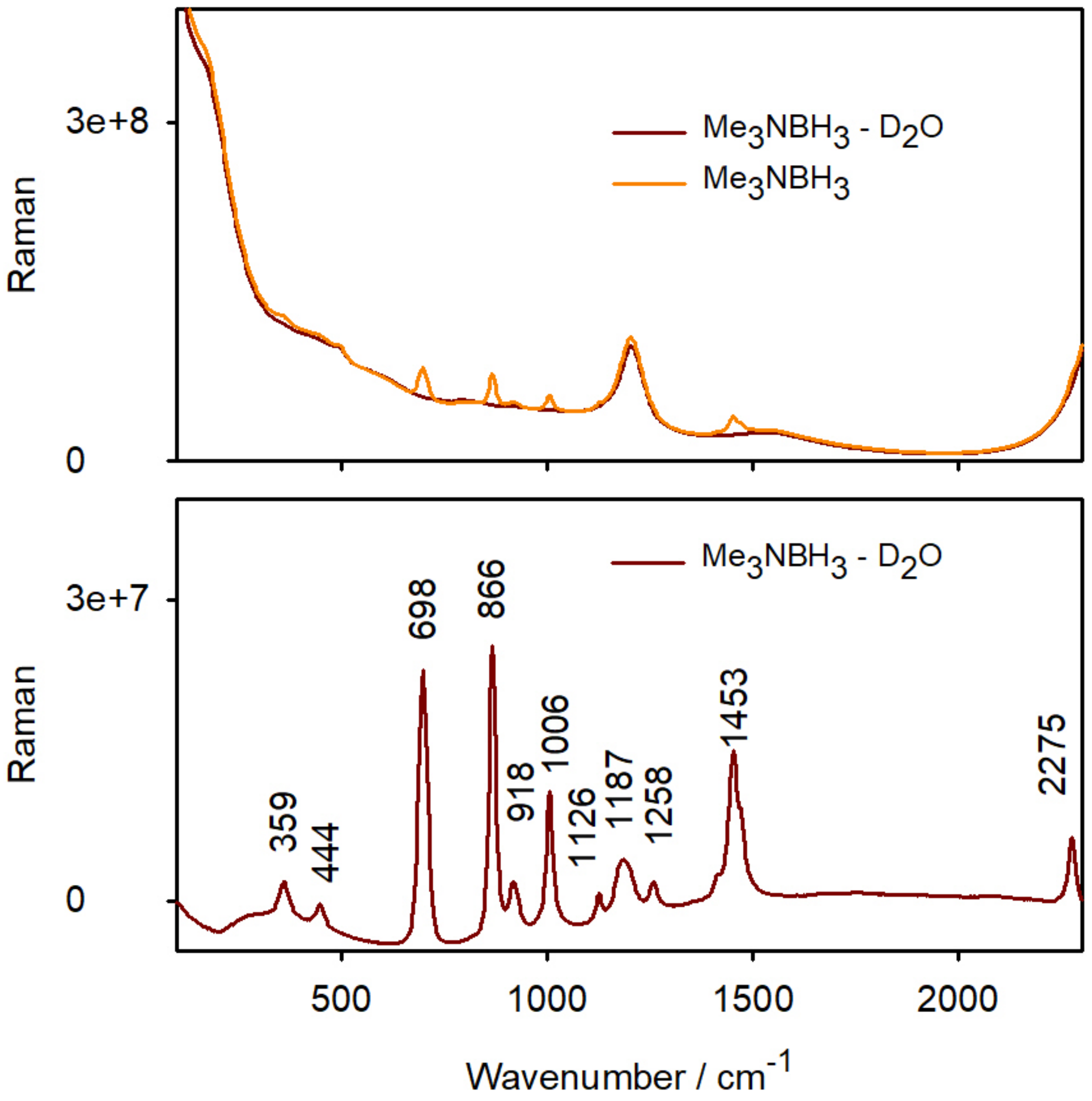

Supplementary Fig. 7 D_2O solution, raw Me_3NBH_3 and D_2O Raman spectra (top), and after subtraction of the D_2O baseline (bottom).

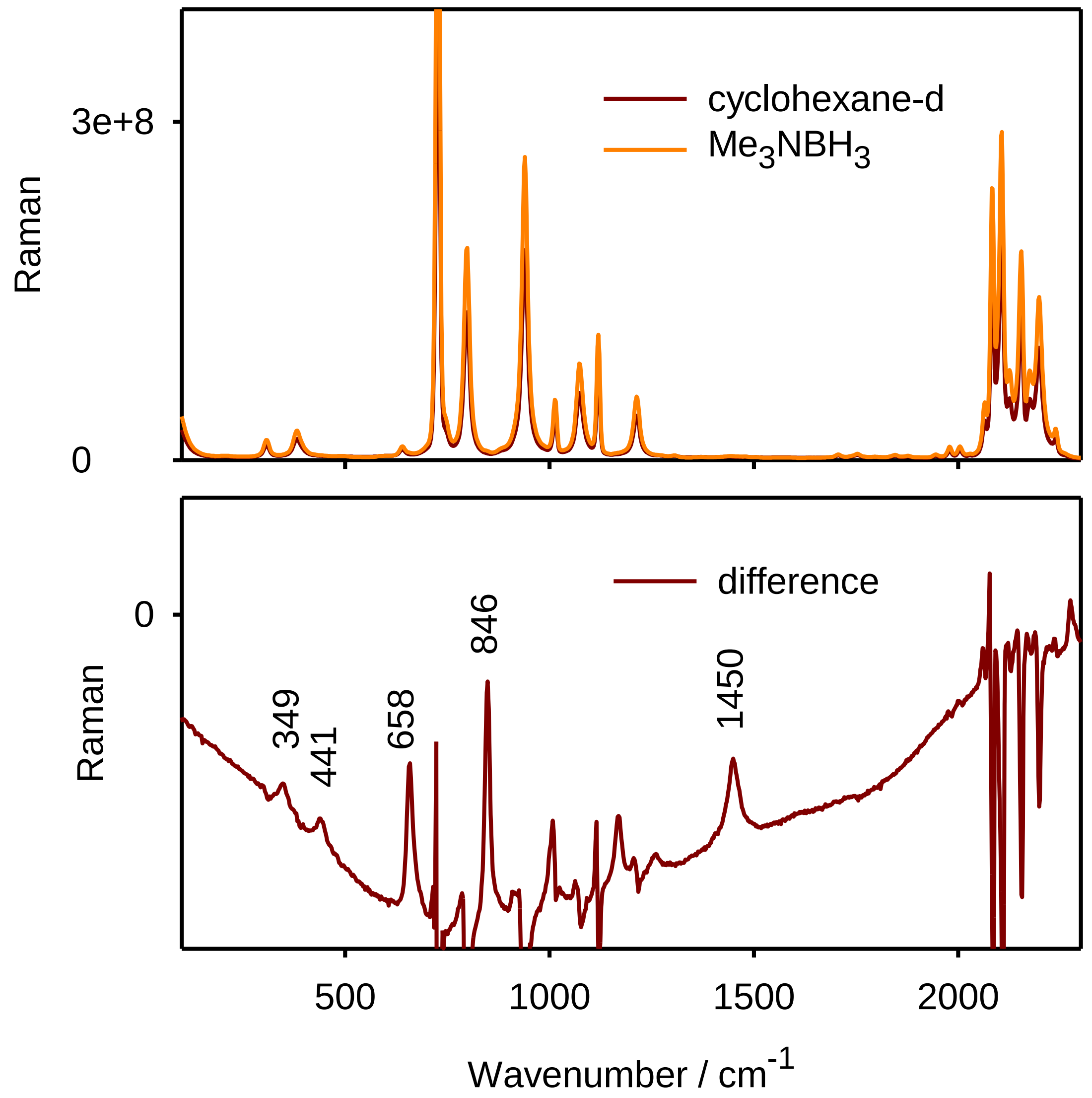

Supplementary Fig. 8 Cyclohexane solution, raw Me_3NBH_3 and cyclohexane Raman spectra (top), and the difference (bottom). Also for other organic solvents the signal was small and only some Me_3NBH_3 bands are could be extracted.

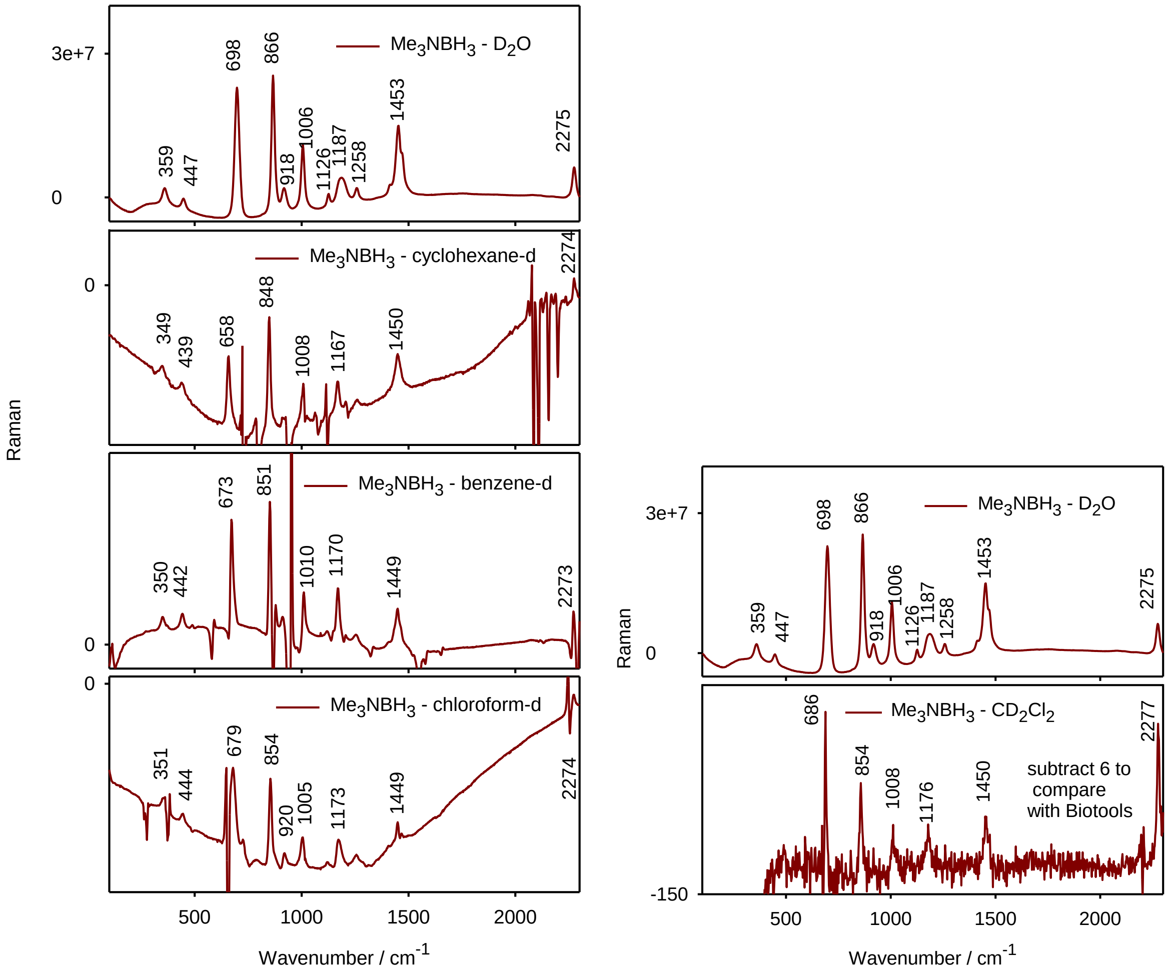

Supplementary Fig. 9 Me_3NBH_3 Raman spectra in D_2O, deuterated cyclohexane, benzene and chloroform (left), and comparison of the spectra in D_2O and dichloromethane (right).

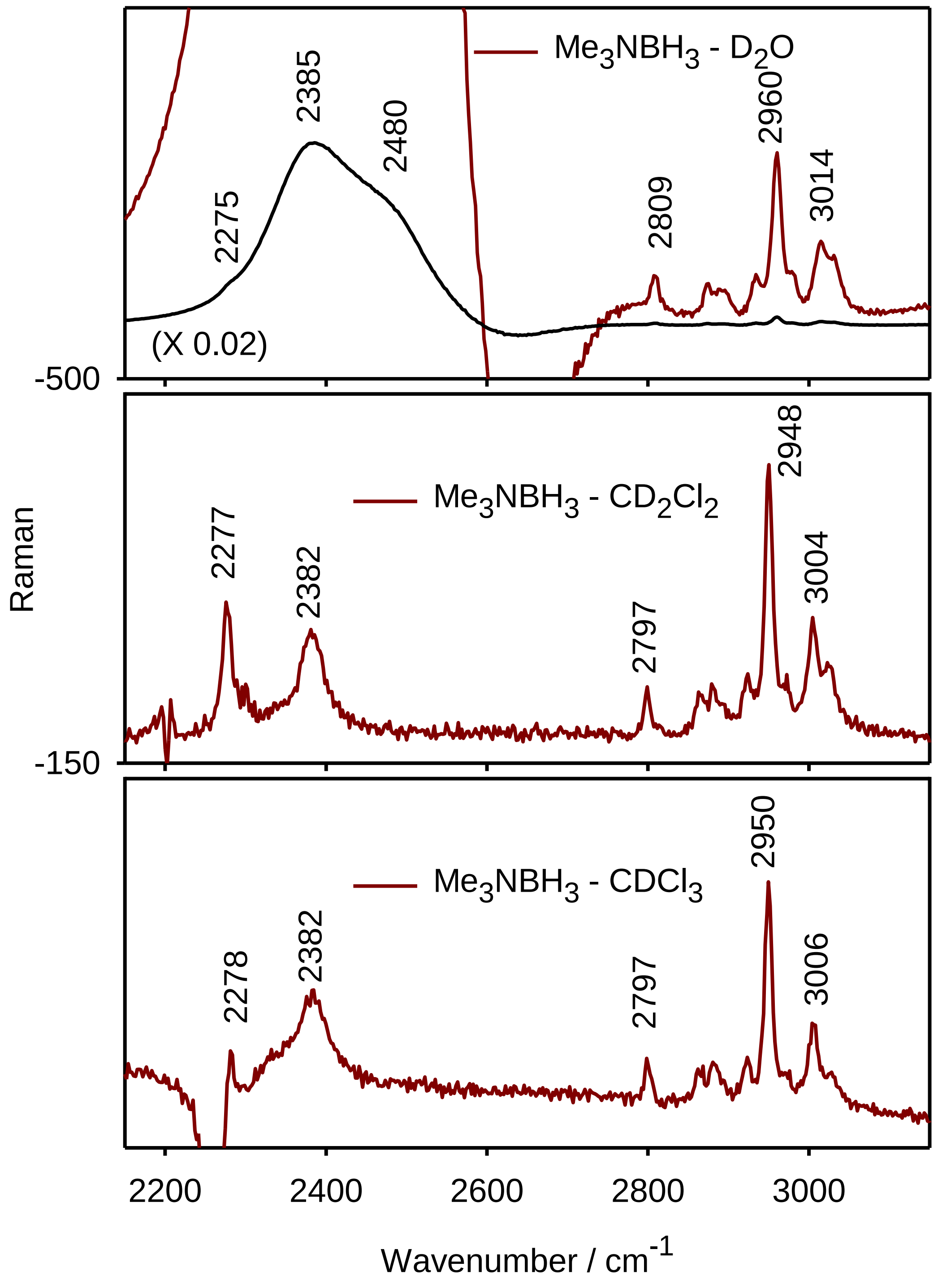

Supplementary Fig. 10 Me_3NBH_3 Raman spectra in deuterated water, dichloromethane and chloroform, the high wavenumber region.

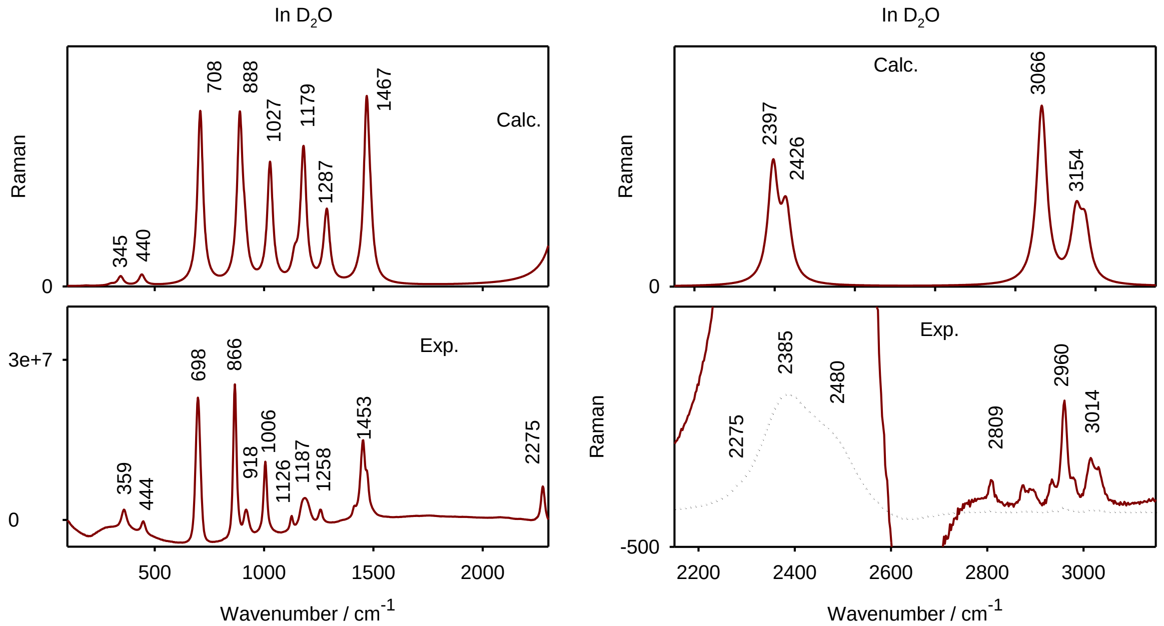

Supplementary Fig. 11 $Me_3N{:}BH_3$ Raman spectra in D_2O, calculation (PBE0-D3/def2-QZVP /COSMO (H_2O)) and experiment, in the mid and high IR regions.

Supplementary References

1. Řezáč, J. *J. Comput. Chem.* **37**, 1230–1237 (2016).
2. Řezáč, J.Cuby – ruby framework for computational chemistry, version 4,
3. TURBOMOLE V7.3 2018, a development of University of Karlsruhe and Forschungszentrum Karlsruhe GmbH, 1989-2007, TURBOMOLE GmbH, since 2007; available from http://www.turbomole.com.
4. Gaussian 09, Revision D.01, Frisch, M. J. et al. Gaussian, Inc., Wallingford CT, 2009.
5. Jamróz, M. H. Vibrational energy distribution analysis (VEDA): Scopes and limitations, *Spectrochimica Acta Part A: Molecular and Biomolecular Spectroscopy* **114**, 220–230 (2013).
6. MOLPRO, version 2010.1, a package of ab initio programs, Werner, H.-J., Knowles, P. J., Knizia, G., Manby, F. R., Schütz, M. and others, see http://www.molpro.net .
7. Neese, F. *Wiley Interdiscip. Rev.: Comput. Mol. Sci*. **2**, 73–78 (2012).
8. Humphrey, W., Dalke, A. & Schulten, K. *J. Mol. Graph*. **14**, 33 (1996).
9. Berendsen, H. J. C., Postma, J. P. M., van Gunsteren, W. F., DiNola, A. & Haak, J. R. *J. Chem. Phys.* **81**, 3684 (1984).
